# Natural vs. Assisted Conception: Sleep and Emotional Health from Pregnancy to Postpartum—An Exploratory Study

**DOI:** 10.3390/jcm14176310

**Published:** 2025-09-06

**Authors:** Olympia Evagorou, Aikaterini Arvaniti, Spyridon Plakias, Nikoleta Koutlaki, Magdalini Katsikidou, Sofia Sfelinioti, Paschalis Steiropoulos, Maria Samakouri

**Affiliations:** 1 Department of Psychiatry, Faculty of Medicine, Democritus University of Thrace, 68100 Alexandroupolis, Greece; oevagoro@med.duth.gr (O.E.); mkatsiki@med.duth.gr (M.K.); sofiasfel@gmail.com (S.S.); msamakou@med.duth.gr (M.S.); 2 Department of Physical Education and Sports, University of Thessaly, 42100 Trikala, Greece; spyros_plakias@yahoo.gr; 3 Department of Obstetrics and Gynaecology, Faculty of Medicine, Democritus University of Thrace, 68100 Alexandroupolis, Greece; nkoutlak@med.duth.gr; 4 Department of Pulmonology, Faculty of Medicine, Democritus University of Thrace, 68100 Alexandroupolis, Greece; pstirop@med.duth.gr

**Keywords:** assisted reproduction, natural conception, infertility stress, sleep disturbances in pregnancy, subjective sleep quality, perinatal mental health

## Abstract

**Background/Objectives**: Sleep plays a key role in female fertility. Sleep disturbances (SDis) during pregnancy are common and may negatively affect maternal health, contributing to an increased risk of perinatal depression and anxiety. **Aim**: The present prospective study aimed to examine the interplay of sleep, anxiety, and depression during the pregnancy and postpartum stages, comparing women who conceived naturally (NC) with those who conceived through assisted reproductive treatment (ART). **Methods**: The study included five timepoints: pre-pregnancy (t0), the end of each trimester (t1–t3), and the postpartum period (t4). SDis were assessed using the Pittsburgh Sleep Quality Index (PSQI), the Athens Insomnia Scale (AIS), the Epworth Sleepiness Scale (ESS), the Fatigue Severity Scale (FFS); perinatal depressive and anxiety symptoms were assessed using the Edinburgh Postnatal Depression Scale (EPDS). Demographic and clinical characteristics were also collected. Given the imbalance in group size and the dispersion of values, a negative binomial regression model with robust variances and Satterthwaite approximation for the degrees of freedom was applied. **Results**: Compared to women with NC (N = 37), those undergoing ART (N = 57) were more likely to be older (*p* < 0.001), married (*p* < 0.001), unemployed (*p* < 0.001), and have a history of thyroid disease (*p* = 0.008). Significant differences between different time points were observed in both NC (N = 37) and successfully conceived ART groups (N = 9) in all sleep, fatigue, and well-being parameters. Notably, at the end of the first trimester (t1), the ART group reported more severe insomnia symptoms (*p* = 0.02). **Conclusions**: SDis are common in pregnancy, but more pronounced during the first trimester among women on ART. These findings highlight the need for early screening and targeted psychological support during perinatal care.

## 1. Introduction

Pregnancy and childbirth, a complex desire that involves conscious and unconscious factors that motivate women to reproduce [1], form a pivotal experience in a woman’s life. They constitute a deep and multidimensional experience that has a profound impact on a woman’s personal development and the formation of her identity [2,3]. Pregnancy constitutes a unique period characterized by profound biological, psychological, and social changes, the impact of which varies among individuals. This experience can be even more profound and emotionally charged for women who face fertility challenges, as the journey toward conception often entails significant physical, emotional, and psychological investment. For these women, pregnancy may represent not only the fulfillment of the desire for motherhood, but also the culmination of a long and demanding process, marked by uncertainty, hope, and resilience [4,5]. From a physiological perspective, pregnancy induces adaptations in the body’s physiology, including the endocrine, hematologic, cardiovascular, respiratory and renal system, to support fetal development and maintain maternal homeostasis [6,7]. Commonly observed symptoms include dizziness, headache, indigestion, low back pain, shortness of breath, nausea and sleep problems [7,8,9].

Regarding sleep during pregnancy, significant disturbances may arise due to the anatomical, metabolic and hormonal changes that occur naturally, which, in turn, can negatively affect perinatal health. Specifically, there are reports of the development of diabetes mellitus, cardiometabolic disorders, gastroesophageal reflux, and hypertension, as well as cases of premature labor and cesarean section, placental abruption, prolonged labor, and fetal death [10,11,12]. Sleep disturbances (SDis) also appear to affect women’s mental health perinatally, as they have been linked to an increased risk of pre- and postnatal depression and anxiety [13,14,15].

Sleep, in parallel, has been proven to play a crucial role in female fertility [16,17]. The secretion of all steroid sex hormones and hormones involved in fertility is synchronized with the circadian rhythm. Melatonin concentration in the follicular fluid protects the oocyte from oxidative stress. Disruptions in the circadian rhythm of gonadotropins are associated with irregular menstrual cycles and infertility [16]. Elevated cortisol levels due to chronic sleep disturbances suppress luteinizing hormone and negatively affect embryo implantation [18]. Sleep disorders also alter thyroid hormone secretion, leading to the dysregulation of the hypothalamic–pituitary–gonadal axis [19], as well as changes in the timing and amplitude of prolactin release, which in turn disrupt follicular development, ovulation, and menstrual cycle regularity [20].

This constitutes a particularly significant field, especially for women who struggle with fertility issues and are undergoing assisted reproductive treatment (ART). SDis in this population have been implicated as the main trigger of psychological distress, which is predominantly manifested through significant anxiety symptoms [21]. Short duration and poor quality of sleep are the most common SDis in women undergoing in vitro fertilization (IVF), and they can occur at all stages of the procedure. Disturbances in sleep quality during IVF have been associated with an increased likelihood of failure of the procedure, which is probably directly related to increased sympathetic nervous system function and impaired hypothalamic–pituitary axis function, which has been observed in sleep deprivation states [22]. Additionally, increased serum ghrelin levels [23] and oxidative stress [24,25], phenomena observed in sleep-restricted conditions, appear to play a crucial role, and are associated with the rate of oocyte maturation and cleavage, oocyte quality, and the fertilization process [26].

The study of sleep quality during pregnancy is an important research field, since numerous studies over time have demonstrated not only the negative impact of poor sleep quality on pregnancy outcomes, but also a strong correlation between SDis and perinatal mental health [22,27,28,29,30,31,32]. Nevertheless, even though sleep during pregnancy can often reflect psychological burden and emotional distress, to date only one study has examined the differences in sleep patterns during pregnancy between women with a history of infertility and those who conceived spontaneously [33].

The aim of this study was to investigate psychological distress during pregnancy by assessing sleep quality, insomnia, daytime sleepiness, fatigue, and symptoms of depression. We compared women who conceived naturally (NC) with women who conceived through assisted reproductive technology (ART), focusing on three key questions: (a) How the demographic and clinical profiles of women in the ART and NC groups differ; (b) How sleep, emotional well-being, and fatigue change from pre-pregnancy to childbirth; and (c) Whether there are significant differences between the groups at each observation point.

## 2. Materials and Methods

### 2.1. Sample and Procedure

Women with NC were recruited at the outpatient clinics of the Gynecology Department of the University General Hospital of Alexandroupolis (UGHA), Greece, where they presented for pregnancy confirmation, routine antenatal follow-up, and delivery at the hospital. Women undergoing ART were recruited during their first appointment at the Assisted Reproduction Unit of UGHA when initiating fertility treatment.

Recruitment and the initial study briefing were conducted by the unit’s attending gynecologist or midwife. Participants were predominantly residents of the Eastern Macedonia and Thrace region, as UGHA is a tertiary university referral center serving a wide geographical catchment area. Eligible participants were women aged 18 years and older with the ability to communicate effectively in Greek. The sample was evaluated in five phases, using five questionnaires:

(t0): The initial contact with women in the NC group occurred upon confirmation of pregnancy. For women in the ART group, the first contact took place when they approached the Assisted Reproduction Unit requesting ART, during which the relevant parameters were evaluated prior to exposure to hormonal therapy within the context of assisted reproduction. For both groups, at this time point, their status at one month prior was assessed retrospectively.

(t1): At the end of the first trimester.

(t2): At the end of the second trimester.

(t3): At the end of the third trimester.

(t4): At the end of the first month of the postpartum period.

After being fully informed about the study objectives and voluntarily agreeing to participate by signing a written informed consent form, all participants were asked to complete a demographic and clinical information form and three baseline questionnaires (Pittsburgh Sleep Quality Index, Athens Insomnia Scale, and Epworth Sleepiness Scale) covering their sleep condition one month prior, which represented their typical sleep state before conception or the initiation of assisted reproduction, respectively. Subsequently, the NC group received a folder containing all the questionnaires, which they were instructed to complete at time points t1, t2, t3, and t4. The completed questionnaires were subsequently returned to the researcher. In order to maintain the time points, the participant was contacted either by telephone or in person. For women undergoing ART, a second contact was made on the day of the pregnancy test. In cases of successful conception, these women were provided with a folder containing the full set of questionnaires, which they were asked to complete at the same time points (t1, t2, t3, t4) as women with NC, and to return to the researcher upon completion. The study was conducted from 2019 to 2024.

### 2.2. Questionnaires

1. Demographic and clinical characteristics
  Participants were asked to answer questions regarding the following:
  (I) Demographic characteristics: (a) Age, (b) Employment status, (c) Marital status and
  (II) Clinical characteristics: (a) History of depression, (b) Family history of mood disorders, (c) Current medical conditions, (d) Current use of psychotropic medication, (e) Current use of thyroid medication, (f) Current use of any other medication prescribed for medical conditions other than psychiatric or thyroid disorders.

2. Pittsburgh Sleep Quality Index (PSQI): a self-reported questionnaire consisting of 19 items evaluating sleep quality during the last month. The total score ranges from 0 to 21, where a score over 5 indicates poor sleep quality [34]. The PSQI is an internationally validated tool that has been extensively used and has been recognized in a wide range of studies conducted in Greece for both the assessment and prediction of sleep disorders in various patient populations [35,36].
3. Athens Insomnia Scale (AIS): a self-reported questionnaire consisting of eight items evaluating the presence and assessing the severity of insomnia. The answers range from 0 to 3 and the score is obtained by summing the scores on each question. The score ranges from 0 to 24 and a total score ≥ 6 indicates insufficient sleep [37].
4. Epworth Sleepiness Scale (ESS): a self-reported questionnaire for the assessment of the degree of daytime sleepiness. It includes the description of eight situations for which the participant is asked to estimate the probability of falling asleep for a reason other than tiredness. Each item corresponds to one possible answer and is scored from 0 to 3. The maximum score is 24 [38].
5. Fatigue Severity Scale (FFS): a self-reported questionnaire for the assessment of daily fatigue, consisting of nine statements that represent the severity of fatigue symptoms during the last 2 weeks. The participant is asked to choose a rating on a scale from 1 to 7, depending on the extent to which they agree with these statements, with 1 corresponding to complete disagreement and 7 to complete agreement. The total score ranges from 9 to 63, and a score above 36 indicates severe fatigue [39].
6. Edinburgh Postnatal Depression Scale (EPDS): a self-reported questionnaire consisting of 10 questions. It investigates the presence of antenatal and postnatal depressive symptomatology during the last week. The possible answers are scored from 0 to 3, with a score ranging from 0 to 30 and a cut-off point of 11/12, according to the Greek validation of the instrument [40].

### 2.3. Ethics

The study was conducted in accordance with the Declaration of Helsinki and approved by the Ethics Committee of the UGHA and by the Ethics and Research Integrity Committee (ERIC) of Democritus University of Thrace (DUTH).

#### Statistical Analysis

To answer the first research question, which concerns the profile of women who visit public hospitals for ART, we conducted statistical tests to determine whether these women differed from those who conceived naturally in the following variables: age, employment, marital status, history of depression, family history of mood disorders, other medical conditions, and the use of psychotropic, thyroid and other medication.

For the continuous variable “age”, the Shapiro–Wilk test, the Kolmogorov–Smirnov test, and the Q–Q plot indicated that the data follow a normal distribution in both groups (NC-ART). Since Levene’s test showed that the assumption of the homogeneity of variances was not met (*p* < 0.001), Welch’s *t*-test was performed to examine whether there was a statistically significant difference in “age” between the two groups. Cohen’s d was used to calculate the effect size, considering the following thresholds: small (0.2), medium (0.5), and large (0.8) [41].

For the categorical variables “employment”, “marital status”, “history of depression”, “family history of mood disorders”, “other medical conditions”, and “use of psychotropic, thyroid and other medication”, Chi-Square tests of independence were conducted to determine whether there was an association between these variables and the type of conception (NC or ART). In cases where the assumptions were not met, i.e., where fewer than 25% of the cells had expected counts of five or less or where no cell had an expected count of zero, Fisher’s exact tests were performed instead. Cramer’s V was used to calculate the effect size, considering the following thresholds: 0.10 indicates a weak association, 0.30 indicates a moderate association, and 0.50 indicates a high association [42].

Next, to examine the changes in the variables PSQI, AIS, ESS, FSS, and EPDS across the different stages of pregnancy, we applied the modern Generalized Linear Mixed Models (GLMMs). These models are suitable for repeated measurements in cases where the assumptions for Repeated Measures ANOVA are not met, while they can also handle unequally spaced time points and missing data, making them very flexible [43,44].

The target variables were PSQI, AIS, ESS, FSS, and EPDS, while the fixed effect in all cases was the interaction term “Conception type” * “TIME”. The variable “Conception type” is categorical with two levels (NC, ART), whereas “TIME” represents the five time points (t0, t1, t2, t3, and t4).

Due to the nature of the data, we used Negative Binomial distribution with a log link, which is appropriate when the target variable represents a count of occurrences with high variance [45]. For the degrees of freedom, we selected Varied across tests (Satterthwaite approximation), because it provides a more accurate estimate of test statistics in cases of smaller and unequal group sizes, as in our study. Additionally, we used robust covariances to handle any violations of model assumptions.

Finally, using Mixed Models, we examined the differences between the two groups (NC vs. ART) at each time point separately, for each of the five variables (PSQI, AIS, ESS, FSS, and EPDS); and the differences in the values of the five variables across the different time points, separately for each group.

## 3. Results

### 3.1. The Demographic and Clinical Profile of the NC and ART Groups and the Differences Between Them

For the first research question, the sample consisted of 37 NC women and 57 women who underwent ART. However, for the second and third research questions, the sample included 37 NC and only 9 ART participants. This discrepancy arises from the fact that the profile was constructed based on all women who presented for assisted reproduction, whereas the variables examined across pregnancy stages included only those ART participants who successfully conceived.

Table 1 presents the demographic and clinical characteristics of the study participants, categorized by type of conception (NC vs. ART). For continuous variables, mean values and standard deviations (SD) are presented, while for categorical variables, the number of participants in each category is reported.

Table 2 presents the statistical comparisons between the NC and ART group, for both continuous and categorical variables.

For the continuous variable “Age”, Welch’s *t*-test indicated a statistically significant difference between the two groups (t = −3.789, *p* < 0.001), with women in the ART group being older. The effect size, measured using Cohen’s d (d = −0.792), suggests a moderate–to–large effect.

Regarding categorical variables, Chi-Square tests of independence revealed significant associations between “Employment” (χ^2^ = 15.103, *p* < 0.001, Cramer’s V = 0.401) and “Marital Status” (χ^2^ = 16.733, *p* < 0.001, Cramer’s V = 0.422), with women in the ART group being more likely to be married and less likely to be employed. Both associations demonstrated a moderate to high effect size based on Cramer’s V values.

Additionally, significant associations were observed for “Medical Conditions” (χ^2^ = 12.488, *p* < 0.001, Cramer’s V = 0.364), “Psychotropic Medication” (*p* = 0.008, Cramer’s V = 0.294), and “Thyroid Medication” (χ^2^ = 6.955, *p* = 0.008, Cramer’s V = 0.272), all indicating moderate associations with the type of conception.

Therefore, women in the ART group were significantly older, more likely to be married, less likely to be employed, and showed higher rates of medical conditions and thyroid medication use, but they were significantly less likely to report the use of psychotropic medication compared to women in the NC group.

Conversely, no significant associations were found for “History of Depression” (*p* = 0.078, Cramer’s V = 0.182), “Family History of Mood Disorders” (*p* = 0.693, Cramer’s V = 0.041), and “Other Medication” (*p* = 0.153, Cramer’s V = 0.195), suggesting a weak or no relationship with the type of conception.

### 3.2. Changes in Sleep, Emotional Well-Being and Fatigue Indices During Pregnancy and the Postpartum Period

Table 3 presents the mean and SD values for PSQI, AIS, ESS, FSS, and EPDS across five time points (t0–t4) separately for the NC group, ART group, and the total sample groups. For FSS and EPDS, there are no measurements at time point t0.

### 3.3. Differences Between the NC and ART Groups and Changes in Psychological and Physiological Measures at Each Stage (t0–t4)

Table 4 presents the adjusted *p*-values for the comparisons between the NC and ART conception groups across the five time points (t0–t4) for PSQI, AIS, ESS, FSS, and EPDS. A statistically significant difference was found in AIS at t1 (*p* = 0.02), indicating that women in the ART group have statistically significantly higher values compared to those in the NC group at this time point. No other variables showed significant differences between groups at any time point after multiple testing adjustments (all other *p*-values > 0.05). The detailed results of all tests are shown in Table S1 of the Supplementary Material.

**Figure 1:**
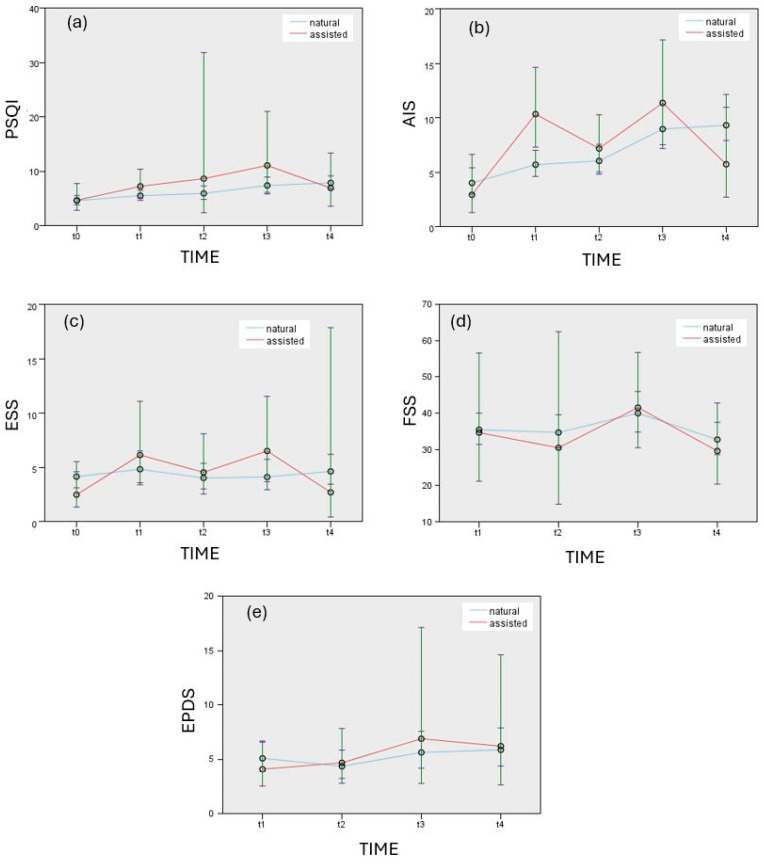
Estimated means charts for significant effects (*p* < 0.05) showing changes in PSQI (**a**), AIS (**b**), ESS (**c**), FSS (**d**), and EPDS (**e**) scores over time (t0–t4) in natural and assisted conception groups.

Figure 1 illustrates the changes in PSQI (a), AIS (b), ESS (c), FSS (d), and EPDS (e) scores across the five time points (t0–t4) for the NC and ART groups. Error bars represent 95% confidence intervals.

**Figure 2:**
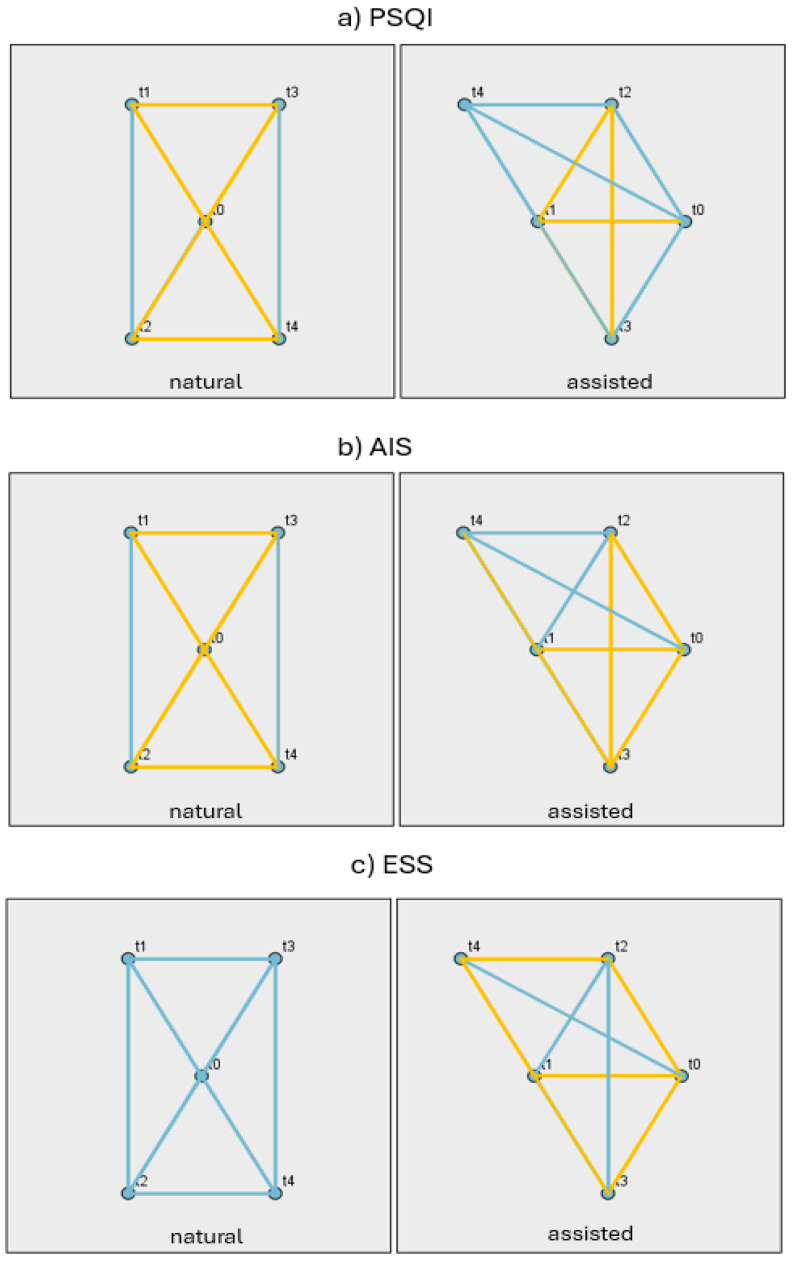
Pairwise contrasts of PSQI (**a**), AIS (**b**), and ESS (**c**) across time points (t0–t4) for natural and assisted conception groups.

Figure 2 displays the pairwise contrasts between time points (t0–t4) for PSQI, AIS, and ESS, separately for the NC and ART groups. Significant contrasts (*p* < 0.05) are highlighted in gold, whereas non-significant contrasts are blue. From this figure, it is evident that only in the NC group for the ESS variable does a consistent pattern emerge, as there are no statistically significant differences among the five time points. On the contrary, in the PSQI and AIS variables, as well as ESS in the ART group, there is a statistically significant difference between time points t0 and t1. The detailed test results are presented in Tables S2–S4 of the Supplementary Material.

**Figure 3:**
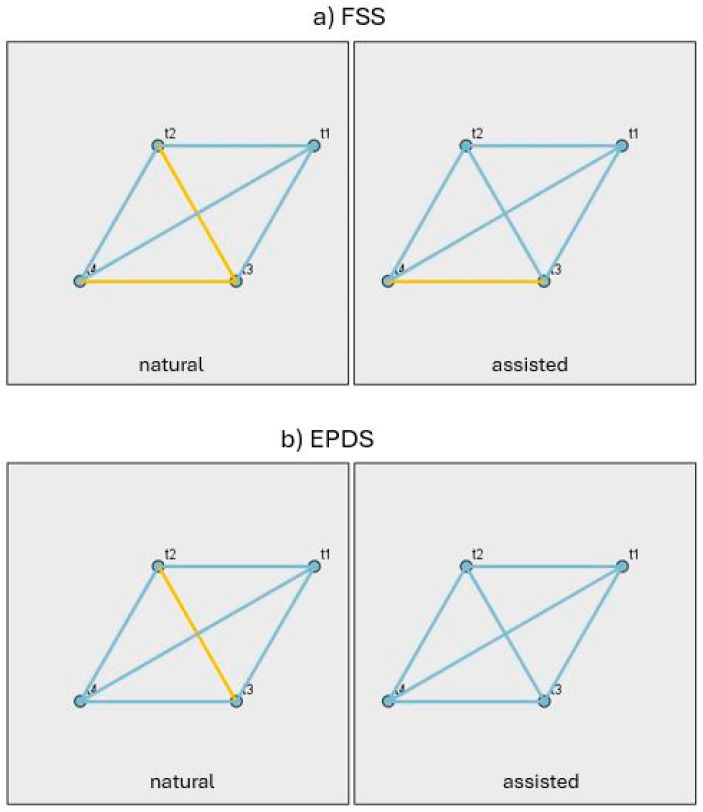
Pairwise contrasts of FSS (**a**) and EPDS (**b**) across time points (t1–t4) for natural and assisted conception groups.

Figure 3 illustrates the pairwise contrasts between time points (t1–t4) for FSS and EPDS, separately for the NC and ART groups. Significant contrasts (*p* < 0.05) are highlighted in gold, while non-significant contrasts are blue. From this figure, it is evident that for none of the two variables, in either group, is there a statistically significant difference between t1 and the other time points (t2, t3, t4). Similarly, no statistically significant difference is observed between t2 and t4. In contrast, a statistically significant difference is found between t2 and t3 in the NC group for the variables FSS and EPDS. Finally, a statistically significant difference is observed between t3 and t4 in both groups for FSS. The detailed test results are presented in Table S5 of the Supplementary Material.

## 4. Discussion

This study offers a comprehensive perspective on the psychological burden experienced by women undergoing fertility treatments and pregnancy, across consecutive stages, including the ART procedure, pregnancy, and the postpartum period, focusing on sleep quality, emotional distress and fatigue. By evaluating the NC group of women and those on ART over time, both intra-individual changes during the perinatal period and intra-group differences depending on the type of conception can be investigated. Changes in sleep patterns, along with changes in mood status, were observed at many stages during pregnancy.

The study revealed some notable differences in terms of demographic and social characteristics between the NC and ART groups. Women in the ART group were significantly older, were more likely to be married and unemployed, and had higher rates of medical conditions and use of thyroid medication, but were significantly less likely to report psychotropic medication use compared to women in the NC group.

The fact that women who chose ART were older than those with NC is consistent with studies showing a decrease in fertility with increasing age [46,47,48,49]. The increased maternal age in this group may be partially attributed to the low success rate of ART deliveries (20–30%), primarily due to the high incidence of miscarriage [50]. It is possible that these women are of advanced maternal age because they underwent several unsuccessful ART attempts or experienced pregnancy loss before achieving a successful outcome. It could also be attributed to a woman’s delay in childbearing due to wanting to first ensure professional advancement [51,52,53] and financial stability [54,55,56]. This contradicts the next finding of the research, namely that a significant proportion of women who resort to ART appear to be outside the labor market, compared to women with NC. This phenomenon could be explained through psychosocial mechanisms: women who are not working may seek meaning, self-fulfillment or social recognition through motherhood [57,58], while working women probably derive part of this satisfaction from their professional role. In addition, it is worth noting that the phenomenon may also reflect social expectations or stereotypes, which encourage women who are outside the labor market to turn more intensely towards motherhood.

An additional finding that emerged from our study was that women with ART were more likely to be married, compared to women with NC. This may reflect a more conscious parenthood, in a more integrated family context, compared to unmarried couples, in whom the pregnancy may not have been planned [59]. Moreover, married couples may regard having a child as an important step toward completing their family, with this desire often being stronger [60] in this group.

A further noteworthy finding concerns the greater likelihood of women undergoing ART to have a medical condition, such as thyroid or autoimmune disease, and the greater likelihood for these women to be receiving treatment for thyroid disorder. This finding is also consistent with the existing literature, which suggests that thyroid disease affects folliculogenesis, fertilization and implantation [61,62,63].

Regarding the findings on sleep patterns and their changes during pregnancy, changes were observed at almost all stages, from conception to the postpartum period. A statistically significant difference between the two populations under examination was observed on the AIS, at time point t1. Differences were also observed at other time points, such as at t3; however, these were not statistically significant, possibly due to the small sample size. It appears that women in the ART group experience insomnia, and its daily consequences, to a greater extent compared to women in the NC group, mainly at the end of the first and third trimesters. Although SDis are expected and observed naturally during pregnancy [64], they seem to affect the ART group at a greater extent, possibly reflecting the greater anxiety that this group may have during these two periods, meaning initially, in the first trimester, a period during which the pregnancy may not develop normally, and the risk of spontaneous abortion is high [65,66]; and at the end of the third trimester, a period shortly before these women’s deep longing for motherhood is finally fulfilled. From this follows the next observation, the improvement of the symptom of insomnia, according to AIS in ART group in the first month of the postpartum period, in which, due to the needs of the newborn (feeding and frequent night waking), women are expected to present higher scores of insomnia and daytime sleepiness and fatigue. As the scale reflects the subjective feeling of the women, it is possible that these women do not consider the consequences of insomnia to be significant, in view of the achievement of having a child. The same applies to measurements on the other scales (PSQI, ESS, FSS, EPDS), where, although an adverse change is observed in sleep patterns, mood status, anxiety and daytime fatigue, all measurements improve postnatally, in this group individually but also compared to the NC group. Although the difference between the two groups is not statistically significant, possibly due to the small sample size, it is possible that in the ART group, after birth, the anxiety of pregnancy and of achieving motherhood subsides, and the event overcomes the challenges and the emotional burden.

SDis were observed in both groups, consistent with the existing literature regarding SDis during pregnancy [64,67]. While in the NC group, the variation was observed to be more linear, in the ART group, it appears to be more pronounced, mainly between values from the beginning of pregnancy and the first trimester (t1), between the second (t2) and third (t3) trimesters, and between the third trimester (t3) and the postpartum period (t4). Cautiously, due to the small sample size, this can lead to the conclusion that SDis during pregnancy in women after ART appear to be more severe. There may be multiple interpretations of this conclusion. First, the association between emotional well-being and sleep has been extensively acknowledged [68,69,70,71,72]. Anxiety about the normal progression of pregnancy, its completion and ultimately the birth of a child, could explain this observation and the greater deviation in the graphs of the first and third trimesters in women in the ART group. Furthermore, the greater fluctuations could be due to the hormonal therapy that women in the ART group underwent, considering the effect of these hormones on sleep patterns and daytime sleepiness [22]. Regarding the parameters for which data were available both for the month prior to pregnancy (t0) and for t1 (PSQI, AIS, ESS), they all show statistically significant differences between the two time points for each group individually, except for ESS in women in the NC group. Therefore, upon entering pregnancy, all women appear to experience changes in their sleep patterns. However, a significant increase in daytime sleepiness is observed only in the ART group. This may indicate that psychological distress in women who conceived via ART is more pronounced (69), potentially due to the previously discussed contributing factors. However, although the daytime sleepiness (ESS) and insomnia (AIS) scales appear to worsen in the first trimester, it appears that, in the corresponding period, women in the ART group do not complain of daytime fatigue, with the FSS-scale notably improving during this time. This, again, could reflect the conscious tolerance of these symptoms by the women in the ART group, due to their great desire and eventual achievement of pregnancy. It could also be attributed to daytime naps, the presence and frequency of which were not measured in this study, as most women in the ART group, being unemployed, would likely have had a greater opportunity for napping.

A statistically significant difference is found between t2 and t3 in the NC group for the variables FSS and EPDS, a pattern that was not observed in the ART group. While this discrepancy could, in part, be attributed to the smaller sample size of the ART group, it may also reflect a higher degree of psychosocial strain among women in the NC group, given that a larger proportion of them are employed compared to those in the ART group. This, in combination with the presence of SDis, may help explain the increased levels of daytime fatigue reported in this group. The changes observed in EPDS scores may potentially be attributed to the parameter of fatigue, as fatigue, both in its intensity and subjective perception, has been shown to negatively influence mood and increase anxiety-related symptoms [73,74]. Finally, a statistically significant difference is observed between t3 and t4 in both groups for FSS, with the scale showing improvement during the first month of the postpartum period in both groups. This observation may possibly be attributed to the postpartum regression of the somatic and physiological changes that occur in women during pregnancy, which impose a physical burden and consequently contribute to daytime fatigue [75].

To our knowledge, this study is the first to present a comprehensive investigation of the specific demographic and clinical characteristics of women undergoing ART, in relation to women with NC, and the second to evaluate the differences in sleep quality and patterns between these two populations. However, several limitations must be acknowledged. The small sample size of the group of the ART participants who successfully conceived (*n* = 9) limits generalizability and reduces statistical power, particularly for between–group comparisons. Although significant patterns emerged, particularly in early pregnancy, the findings should be interpreted with caution. Nonetheless, despite the small sample size, the use of a Negative Binomial model with robust covariances and the Satterthwaite approximation for degrees of freedom helped mitigate some of the statistical limitations associated with unequal group sizes and high data dispersion. Another limitation is the presence of baseline demographic and clinical differences between the ART and NC groups (e.g., age, marital status, employment, thyroid disease history). Given the limited sample size, it was not feasible to adjust for these potential confounders through multivariable analyses, and therefore any residual confounding may have influenced the observed associations. In addition, the use of self-report questionnaires may introduce response bias, since objective measures of sleep, such as actigraphy, were not included. Furthermore, due to the small sample size, it was not possible to implement more complex longitudinal models (e.g., random slopes or cross-lagged approaches) that could capture individual trajectories and better assess the temporal interplay between sleep disturbances and psychological symptoms. Finally, while multiple validated instruments were used, overlapping symptoms (e.g., SDis and fatigue) may complicate interpretation. Future research with larger, more balanced sample sizes and objective assessments is needed to confirm these findings.

Despite these limitations, the study provides valuable preliminary data highlighting the psychosocial vulnerability of pregnant women who conceived through ART, particularly in early pregnancy, and supports the importance of individualized mental health monitoring throughout the perinatal period. The hypothesized psychological mechanisms underlying the observed differences, such as heightened anxiety, strong maternal desire, and subjective tolerance, may have direct implications for clinical practice. These factors can shape not only the perception and reporting of sleep and mood disturbances during pregnancy, but also the capacity to cope with them, thereby influencing overall maternal well-being. Recognizing these mechanisms underscores the need for systematic screening of pregnant women, particularly those who conceived through ART, for sleep and psychological symptoms as part of routine antenatal care. Early identification could facilitate timely, individualized interventions, including psychoeducation, stress reduction strategies, and sleep hygiene counseling, which address both the physiological and psychological aspects of pregnancy. Such an approach may enhance maternal quality of life and potentially improve pregnancy outcomes.

## 5. Conclusions

Women experiencing fertility challenges, including those undergoing ART, represent a particularly vulnerable population, having invested significant physical and emotional resources in the pursuit of parenthood. While alterations in sleep patterns are commonly anticipated during pregnancy, in this subgroup, they warrant careful attention and should not be regarded as a secondary concern.

These women often present distinct demographic and clinical profiles that may interact with psychological stressors in complex ways. Entering pregnancy with a heightened psychological burden, their anxiety regarding pregnancy maintenance and outcomes is frequently intensified. Given that emotional distress frequently manifests as SDis, routine assessment of sleep, implementation of preventive strategies, and timely therapeutic interventions should be integral components of perinatal care in this population.

## Figures and Tables

**Table 1:** Demographic and clinical characteristics of participants.

Variable	Type of Conception					
	NC		ART		Total	
	Continuous					
	Mean	SD	Mean	SD	Mean	SD
Age	32.444	4.116	35.964	4.643	34.571	4.745
	Categorical					
	Yes	No	Yes	No	Yes	No
Employment	35	2	33	24	68	26
Married	24	13	55	2	79	15
History of depression	6	31	3	54	9	85
Family history of mood disorders	7	30	9	48	16	78
Medical conditions	5	32	28	29	33	61
Psychotropic medication	5	32	0	57	5	89
Thyroid medication	4	33	20	37	24	70
Other medication (prescribed for medical conditions other than psychiatric or thyroid disorders)	0	36	5	50	5	86

NC: natural conception, ART: assisted reproductive treatment.

**Table 2:** Statistical comparisons of demographic and clinical characteristics between women with NC and ART.

Variable	Test	df	*p*	Effect Size
	Continuous			
	**t**	**df**	***p***	**Cohen’s d**
Age	−3.789	81.111	<0.001	−0.792
	Categorical			
	**x^2^**	**df**	***p***	**Cramer’s V**
Employment	15.103	1	<0.001	0.401
Married	16.733	1	<0.001	0.422
History of depression	3.109	1	0.078	0.182
Family history of mood disorders	0.156	1	0.693	0.041
Medical conditions	12.488	1	<0.001	0.364
Psychotropic medication			0.008	0.294
Thyroid medication	6.955	1	0.008	0.272
Other medication (prescribed for medical conditions other than psychiatric or thyroid disorders)			0.153	0.195

NC: natural conception, ART: assisted reproductive treatment.

**Table 3:** Descriptive statistics (Mean, SD) of PSQI, AIS, ESS, FSS, and EPDS across different time points (t0–t4) for NC and ART groups.

Conception Type	Variable	Time									
		t0		t1		t2		t3		t4	
		Mean	SD	Mean	SD	Mean	SD	Mean	SD	Mean	SD
NC	PSQI	4.89	2.90	5.92	3.14	6.59	3.81	8.23	4.53	8.51	3.78
	AIS	4.53	4.09	6.25	3.81	6.89	4.68	10.17	6.30	10.03	4.88
	ESS	4.97	3.92	5.91	4.47	5.11	3.84	5.29	4.45	5.54	4.08
	FSS			36.97	12.74	36.67	14.31	42.09	15.71	34.29	13.29
	EPDS			6.32	5.99	5.53	5.60	6.66	6.21	6.09	5.20
ART	PSQI	5.00	4.00	7.38	3.25	8.75	2.71	11.57	3.41	7.29	4.50
	AIS	3.56	4.61	10.50	4.54	8.00	4.38	13.43	7.48	7.00	7.01
	ESS	2.78	2.39	6.75	4.13	5.00	2.51	7.29	4.54	3.00	1.67
	FSS			38.13	17.72	33.75	16.12	43.29	18.02	32.17	18.67
	EPDS			5.00	4.28	6.25	6.90	8.43	9.64	7.00	5.18
Total	PSQI	4.91	3.10	6.18	3.17	7.00	3.70	8.79	4.51	8.31	3.88
	AIS	4.33	4.16	7.02	4.23	7.09	4.59	10.71	6.53	9.59	5.25
	ESS	4.53	3.75	6.07	4.37	5.09	3.61	5.62	4.47	5.17	3.92
	FSS			37.18	13.52	36.14	14.51	42.29	15.89	33.98	13.93
	EPDS			6.09	5.70	5.66	5.78	6.95	6.78	6.22	5.15

NC: natural conception, ART: assisted reproductive treatment, PSQI: Pittsburgh Sleep Quality Index, AIS: Athens Insomnia Scale, ESS: Epworth Sleepiness Scale, FSS: Fatigue Severity Scale, EPDS: Edinburgh Postnatal Depression Scale.

**Table 4:** Adjusted *p*-values for comparisons between NC and ART groups at each time point (t0–t4) for PSQI, AIS, ESS, FSS, and EPDS.

Contrast	TIME	PSQI	AIS	ESS	FSS	EPDS
NC-ART	t0	0.959	0.402	0.079		
	t1	0.15	0.02	0.437	0.908	0.383
	t2	0.131	0.419	0.608	0.542	0.807
	t3	0.095	0.35	0.19	0.825	0.659
	t4	0.541	0.104	0.056	0.592	0.896

NC: natural conception, ART: assisted reproductive treatment, PSQI: Pittsburgh Sleep Quality Index, AIS: Athens Insomnia Scale, ESS: Epworth Sleepiness Scale, FSS: Fatigue Severity Scale, EPDS: Edinburgh Postnatal Depression Scale.

## Data Availability

The data supporting the findings of this study are available from the corresponding author upon reasonable request.

## Supplementary Materials

The following supporting information can be downloaded at: https://www.mdpi.com/article/10.3390/jcm14176310/s1, Table S1: Pairwise contrasts for PHQI, AIS, ESS, FSS, and EPDS across time points (t0–t4) comparing NC vs ART conception groups. Contrast estimates, standard errors, t-values, degrees of freedom (df), adjusted significance (Adj. Sig.), and 95% confidence intervals (CI) are reported. Table S2: Pairwise contrasts for PHQI (for both NC and ART groups) comparing time points (t0–t4). Contrast estimates, standard errors, t-values, degrees of freedom (df), adjusted significance (Adj. Sig.), and 95% confidence intervals (CI) are reported. Table S3: Pairwise contrasts for AIS (for both NC and ART groups) comparing time points (t0–t4). Contrast estimates, standard errors, t-values, degrees of freedom (df), adjusted significance (Adj. Sig.), and 95% confidence intervals (CI) are reported. Table S4: Pairwise contrasts for ESS (for both NC and ART groups) comparing time points (t0–t4). Contrast estimates, standard errors, t-values, degrees of freedom (df), adjusted significance (Adj. Sig.), and 95% confidence intervals (CI) are reported. Table S5: Pairwise contrasts for FSS, and EPDS (for both NC and ART groups) comparing time points (t1–t4). Contrast estimates, standard errors, t-values, degrees of freedom (df), adjusted significance (Adj. Sig.), and 95% confidence intervals (CI) are reported.

## Abbreviations

The following abbreviations are used in this manuscript:

SDis	Sleep Disturbances
NC	Naturally Conceived
ART	Assisted Reproductive Treatment
UGHA	University General Hospital of Alexandroupolis
PSQI	Pittsburgh Sleep Quality Index
AIS	Athens Isomnia Scale
ESS	Epworth Sleepiness Scale
FSS	Fatigue Se`verity Scale
EPDS	Edinburgh Postnatal Depression Scale
