# Supplementary material for: Natural vs. Assisted Conception: Sleep and Emotional Health from Pregnancy to Postpartum—An Exploratory Study
Source: J Clin Med. 2025 Sep 6;14(17):6310. doi: 10.3390/jcm14176310 (PMC12429457; doi:10.3390/jcm14176310)
Supplement: Supplementary file 1 [file jcm-14-06310-s001.zip › jcm-3804024-supplementary.pdf]

Table S1. Pairwise contrasts for PHQI, AIS, ESS, FSS, and EPDS across time points (t0–t4) comparing NC vs ART conception groups. Contrast estimates, standard errors, t-values, degrees of freedom (df), adjusted significance (Adj. Sig.), and 95% confidence intervals (CI) are reported.

Contrast	Variable	TIME	Contrast Estimate	Std. Error	t	df	Adj. Sig.	95% CI Lower	95% CI Upper
NC-ART	PHQI	t0	0.064	1.233	0.052	38	0.959	-2.433	2.561
		t1	1.725	1.043	1.654	6	0.15	-0.836	4.286
		t2	2.725	0.967	2.817	2	0.131	-2.393	7.844
		t3	3.688	1.356	2.719	2	0.095	-1.406	8.782
		t4	-0.963	1.438	-0.669	4	0.541	-5.025	3.1
	AIS	t0	-1.085	1.264	-0.859	18	0.402	-3.744	1.573
		t1	4.646	1.572	2.956	7	0.02	0.977	8.315
		t2	1.147	1.396	0.821	26	0.419	-1.724	4.017
		t3	2.398	2.546	0.942	67	0.35	-2.684	7.479
		t4	-3.587	2.014	-1.781	10	0.1	-8.051	0.876
	ESS	t0	-1.67	0.916	-1.824	28	0.079	-3.548	0.207
		t1	1.309	1.602	0.817	8	0.437	-2.385	5.003
		t2	0.523	0.956	0.547	5	0.608	-1.947	2.994
		t3	2.404	1.673	1.437	8	0.19	-1.484	6.291
		t4	-1.927	0.855	-2.253	8	0.056	-3.922	0.067
	FSS	t1	-0.811	6.681	-0.121	5	0.908	-17.583	15.962
		t2	-4.226	6.164	-0.686	3	0.542	-23.753	15.301
		t3	1.566	7.034	0.223	58	0.825	-12.516	15.647
		t4	-3.159	5.844	-0.541	41	0.592	-14.959	8.641
	EPDS	t1	-0.998	1.115	-0.895	17	0.383	-3.349	1.353
		t2	0.326	1.327	0.246	31	0.807	-2.38	3.032
		t3	1.262	2.749	0.459	8	0.659	-5.095	7.619
		t4	0.339	2.537	0.134	12	0.896	-5.178	5.857

NC: natural conception, ART: assisted reproductive treatment, PHQI: Pittsburgh Sleep Quality Index, AIS: Athens Insomnia Scale, ESS: Epworth Sleepiness Scale, FSS: Fatigue Severity Scale, EPDS: Edinburgh Postnatal Depression Scale

Table S2. Pairwise contrasts for PHQI (for both NC and ART groups) comparing time points (t0–t4). Contrast estimates, standard errors, t-values, degrees of freedom (df), adjusted significance (Adj. Sig.), and 95% confidence intervals (CI) are reported.

Variable	Group	TIME Comparison	Contrast Estimate	Std. Error	t	df	Adj. Sig.	95% CI Lower	95% CI Upper
PHQI	NC	t0 - t1	-0.897	0.441	-2.034	119	0.044	-1.771	-0.024
		t0 - t2	-1.318	0.548	-2.406	3	0.088	-2.976	0.34
		t0 - t3	-2.771	0.64	-4.33	5	0.006	-4.379	-1.163
		t0 - t4	-3.264	0.651	-5.017	73	<0.001	-4.56	-1.967
		t1 - t2	-0.421	0.447	-0.941	4	0.401	-1.674	0.833
		t1 - t3	-1.874	0.574	-3.265	7	0.013	-3.222	-0.525
		t1 - t4	-2.366	0.554	-4.271	48	<0.001	-3.48	-1.253
		t2 - t3	-1.453	0.367	-3.955	53	<0.001	-2.19	-0.716
		t2 - t4	-1.945	0.444	-4.382	5	0.009	-3.12	-0.771
		t3 - t4	-0.493	0.487	-1.011	5	0.356	-1.724	0.738
	ART	t0 - t1	-2.558	1.028	-2.488	6	0.047	-5.07	-0.047
		t0 - t2	-3.98	0.729	-5.458	1	0.159	-21.022	13.062
		t0 - t3	-6.395	1.024	-6.245	1	0.107	-20.495	7.705
		t0 - t4	-2.237	1.357	-1.648	4	0.183	-6.196	1.722
		t1 - t2	-1.421	0.637	-2.23	16	0.041	-2.774	-0.069
		t1 - t3	-3.837	1.066	-3.6	7	0.008	-6.339	-1.335
		t1 - t4	0.321	1.206	0.266	24	0.792	-2.171	2.813
		t2 - t3	-2.415	0.746	-3.236	19	0.004	-3.976	-0.855
		t2 - t4	1.743	1.416	1.231	10	0.247	-1.428	4.913
		t3 - t4	4.158	1.867	2.227	7	0.06	-0.236	8.553

NC: natural conception, ART: assisted reproductive treatment, PHQI: Pittsburgh Sleep Quality Index, AIS: Athens Insomnia Scale, ESS: Epworth Sleepiness Scale, FSS: Fatigue Severity Scale, EPDS: Edinburgh Postnatal Depression Scale

Table S3. Pairwise contrasts for AIS (for both NC and ART groups) comparing time points (t0–t4). Contrast estimates, standard errors, t-values, degrees of freedom (df), adjusted significance (Adj. Sig.), and 95% confidence intervals (CI) are reported.

Variable	Group	TIME Comparison	Contrast Estimate	Std. Error	t	df	Adj. Sig.	95% CI Lower	95% CI Upper
AIS	NC	t0 - t1	-1.681	0.513	-3.277	136	0.001	-2.695	-0.666
		t0 - t2	-2.023	0.646	-3.133	52	0.003	-3.319	-0.727
		t0 - t3	-4.959	0.928	-5.343	67	<0.001	-6.811	-3.106
		t0 - t4	-5.306	0.783	-6.773	35	<0.001	-6.896	-3.716
		t1 - t2	-0.342	0.539	-0.635	30	0.53	-1.444	0.759
		t1 - t3	-3.278	0.986	-3.324	34	0.002	-5.282	-1.273
		t1 - t4	-3.626	0.775	-4.68	38	<0.001	-5.195	-2.057
		t2 - t3	-2.935	0.765	-3.838	63	<0.001	-4.464	-1.407
		t2 - t4	-3.283	0.696	-4.719	11	0.001	-4.812	-1.754
		t3 - t4	-0.348	0.797	-0.437	19	0.667	-2.014	1.318
	ART	t0 - t1	-7.412	1.757	-4.218	4	0.018	-12.557	-2.267
		t0 - t2	-4.255	0.814	-5.227	10	<0.001	-6.062	-2.448
		t0 - t3	-8.442	1.905	-4.431	70	<0.001	-12.241	-4.643
		t0 - t4	-2.804	1.458	-1.923	18	0.07	-5.866	0.257
		t1 - t2	3.157	1.831	1.724	11	0.114	-0.896	7.21
		t1 - t3	-1.03	2.17	-0.475	9	0.647	-5.949	3.89
		t1 - t4	4.608	2.251	2.047	4	0.118	-1.932	11.147
		t2 - t3	-4.186	1.334	-3.139	138	0.002	-6.824	-1.549
		t2 - t4	1.451	1.672	0.867	6	0.42	-2.673	5.574
		t3 - t4	5.637	2.367	2.382	19	0.028	0.681	10.593

NC: natural conception, ART: assisted reproductive treatment, PHQI: Pittsburgh Sleep Quality Index , AIS: Athens Insomnia Scale, ESS: Epworth Sleepiness Scale, FSS: Fatigue Severity Scale, EPDS: Edinburgh Postnatal Depression Scale

Table S4. Pairwise contrasts for ESS (for both NC and ART groups) comparing time points (t0–t4). Contrast estimates, standard errors, t-values, degrees of freedom (df), adjusted significance (Adj. Sig.), and 95% confidence intervals (CI) are reported.

Variable	Group	TIME Comparison	Contrast Estimate	Std. Error	t	df	Adj. Sig.	95% CI Lower	95% CI Upper
ESS	NC	t0 - t1	-0.676	0.523	-1.291	111	0.199	-1.713	0.361
		t0 - t2	0.128	0.464	0.276	16	0.786	-0.855	1.111
		t0 - t3	0.041	0.651	0.063	18	0.95	-1.325	1.325
		t0 - t4	-0.477	0.6	-0.796	56	0.429	-1.678	0.724
		t1 - t2	0.804	0.539	1.491	38	0.144	-0.288	1.985
		t1 - t3	0.717	0.675	1.061	31	0.297	-0.662	2.096
		t1 - t4	0.199	0.669	0.297	54	0.768	-1.142	1.539
		t2 - t3	-0.087	0.37	-0.235	47	0.815	-0.832	0.658
		t2 - t4	-0.605	0.523	-1.157	27	0.257	-1.678	0.468
		t3 - t4	-0.518	0.602	-0.861	24	0.398	-1.761	1.676
	ART	t0 - t1	-3.655	1.392	-2.627	22	0.015	-6.542	-0.769
		t0 - t2	-2.066	0.849	-2.433	9	0.038	-3.993	-0.138
		t0 - t3	-4.033	1.13	-3.568	13	0.003	-6.475	-1.591
		t0 - t4	-0.22	0.557	-0.396	32	0.695	-1.354	0.913
		t1 - t2	1.59	1.246	1.276	70	0.206	-0.894	4.074
		t1 - t3	-0.378	1.439	-0.262	100	0.794	-3.233	2.478
		t1 - t4	3.435	1.371	2.506	60	0.015	0.693	6.177
		t2 - t3	-1.967	1.319	-1.491	50	0.142	-4.617	0.682
		t2 - t4	1.845	0.783	2.356	30	0.025	0.246	3.444
		t3 - t4	3.813	1.057	3.607	23	0.001	1.627	5.999

NC: natural conception, ART: assisted reproductive treatment, PHQI: Pittsburgh Sleep Quality Index , AIS: Athens Insomnia Scale, ESS: Epworth Sleepiness Scale, FSS: Fatigue Severity Scale, EPDS: Edinburgh Postnatal Depression Scale

Table S5. Pairwise contrasts for FSS, and EPDS (for both NC and ART groups) comparing time points (t1–t4). Contrast estimates, standard errors, t-values, degrees of freedom (df), adjusted significance (Adj. Sig.), and 95% confidence intervals (CI) are reported.

Variable	Group	TIME Comparison	Contrast Estimate	Std. Error	t	df	Adj. Sig.	95% CI Lower	95% CI Upper
FSS	NC	t1 - t2	0.768	1.499	0.513	14	0.616	-2.45	3.987
		t1 - t3	-4.569	2.515	-1.817	25	0.081	-9.749	0.61
		t1 - t4	2.718	1.999	1.36	28	0.185	-1.378	6.813
		t2 - t3	-5.337	1.84	-2.901	60	0.005	-9.018	-1.657
		t2 - t4	1.949	1.781	1.094	36	0.281	-1.662	5.561
		t3 - t4	7.287	1.812	4.022	96	<0.001	3.69	10.883
	ART	t1 - t2	4.184	2.914	1.436	94	0.154	-1.602	9.969
		t1 - t3	-6.945	5.4	-1.286	10	0.226	-18.921	5.031
		t1 - t4	5.066	5.108	0.992	5	0.367	-8.081	18.213
		t2 - t3	-11.129	4.376	-2.543	5	0.055	-22.599	0.341
		t2 - t4	0.882	5.155	0.171	4	0.873	-14.216	15.981
		t3 - t4	12.011	4.802	2.502	66	0.015	2.423	21.599
EPDS	NC	t1 - t2	0.734	0.378	1.94	37	0.06	-0.033	1.501
		t1 - t3	-0.545	0.677	-0.805	39	0.426	-1.914	0.824
		t1 - t4	-0.784	0.854	-0.918	7	0.387	-2.781	1.213
		t2 - t3	-1.279	0.611	-2.094	54	0.041	-2.504	-0.054
		t2 - t4	-1.518	0.79	-1.922	7	0.098	-3.408	0.372
		t3 - t4	-0.239	0.764	-0.313	48	0.756	-1.774	1.296
	ART	t1 - t2	-0.59	0.555	-1.063	15	0.304	-1.771	0.591
		t1 - t3	-2.805	2.631	-1.066	10	0.311	-8.668	3.058
		t1 - t4	-2.122	2.099	-1.011	14	0.329	-6.619	2.376
		t2 - t3	-2.215	2.357	-0.94	9	0.373	-7.584	3.154
		t2 - t4	-1.531	2.483	-0.617	11	0.55	-6.985	3.922
		t3 - t4	0.683	3.688	0.185	13	0.856	-7.286	8.653

NC: natural conception, ART: assisted reproductive treatment, PHQI: Pittsburgh Sleep Quality Index , AIS: Athens Insomnia Scale, ESS: Epworth Sleepiness Scale, FSS: Fatigue Severity Scale, EPDS: Edinburgh Postnatal Depression Scale
